# A New Kind of Meerschaum

**Published:** 1870-01

**Authors:** 


A New Kind of Meerschaum.—Chemistry has developed a new use for potatoes and other vegetables, illustrations of which were seen by visitors at the Paris International Exhibition. If potatoes are peeled, macerated for about thirty-six hours in water, to which eight per cent. of sulphuric acid has been added, well washed with water, dried in blotting paper, and then in hot sand for several days, on plates of chalk or plaster of Paris, which are changed daily, being compressed at the same time, an excellent imitation of meerschaum, answering well for the carver, or any purpose not requiring a high temperature, will be obtained. If water containing three per cent. of soda, instead of eight per cent. of sulphuric acid is used, greater hardness, whiteness, and elasticity will be produced. Turnips may be used instead of potatoes in the production of artificial horn. If carrots are substituted for potatoes, an excellent artificial coral will be presented.—*Drug. Circular.*

